# Multi-agent game theory for precision psychiatry

**DOI:** 10.3389/fpsyt.2026.1963963

**Published:** 2026-09-30

**Authors:** Zhe Sage Chen

**Affiliations:** 1 Department of Psychiatry, New York University Grossman School of Medicine, New York, NY, United States; 2 Department of Neuroscience, Institute for Translational Neuroscience, New York University Grossman School of Medicine, New York, NY, United States; 3 Department of Biomedical Engineering, New York University Tandon School of Engineering, Brooklyn, NY, United States

**Keywords:** autism spectrum disorder, computational phenotype, game theory, precision psychiatry, schizophrenia, social interaction

## Abstract

Psychiatry has learned to measure and map the brain in exquisite detail, yet it still lacks a theory of what happens between minds. Most current frameworks treat cognition as an individual process, studied through static and non-interactive tasks, creating a fundamental mismatch with real-world social behavior, which is dynamic and reciprocal. In restricted psychiatric conditions such as autism spectrum disorder (ASD) and schizophrenia with social phenotypes, core symptoms emerge most clearly during interaction. Here I argue that these differences are better understood as failures of coordination between agents rather than solely deficits within individuals, and further make forward-looking computational predictions based on a game-theoretical perspective. Multi-agent game theory (MAGT) provides a conceptual yet principled framework to model such interactions, capturing how beliefs, strategies, and learning evolve through exchange. This perspective reframes psychiatric disorders involving interactional phenotypes as emergent properties of interacting systems and opens new avenues for mechanistic understanding and intervention. I also discuss the limitations and challenges of this new computational framework as applied to precision psychiatry.

## 1 Introduction

Social interaction is a defining feature of human behavior, revealing how individuals infer, adapt to, and influence others (1). Despite advances in neuroimaging, genomics, and machine learning that have propelled precision psychiatry (2), the field still lacks a mechanistic understanding of social interaction. A central limitation is the prevailing individual-centric paradigm, which isolates cognition from the dynamic, reciprocal environments in which it naturally unfolds. Even in social cognition research, behavior is often studied using static, one-shot tasks, creating a mismatch between laboratory measurements and real-world behavior, which unfolds as a dynamic and reciprocal process among interacting agents.

This limitation is evident in psychiatric conditions with interactional deficits, such as autism spectrum disorder (ASD) and schizophrenia, in which core impairments emerge most clearly during real-time social exchange. ASD affects coordination, joint attention, and turn-taking (3), while schizophrenia alters belief attribution and social inference (4, 5). Yet both are predominantly studied in single-agent settings, obscuring how dysfunction arises through interaction. This raises a fundamental question: are social deficits properties of individuals, or emergent features of interacting agents with differing beliefs and strategies?

Existing computational psychiatry approaches (6)—including neuroeconomics (7–10), hyperscanning (11, 12), interactive reinforcement learning (13, 14), and second-person (interactive) neuroscience (15, 16)—have begun to address this gap but remain fragmented. Although game-based tasks have been applied to the study of the mind (17–20) and to psychiatric research (21–24), most implementations rely on simplified, static paradigms that fail to capture real-world dynamics. Furthermore, very few psychiatric studies have been rooted in formal game-theoretic approaches (24, 25). Here, I propose multi-agent game theory (MAGT), grounded in classical game theory and enriched by multi-agent reinforcement learning (MARL) (26–28), as a unifying framework for precision psychiatry. By modeling behavior as interactions among agents with evolving beliefs and strategies, MAGT shifts the explanatory focus from isolated individuals to adaptive systems.

This multi-agent perspective reframes social dysfunction as an emergent property of interaction and provides a principled foundation for measurement, theory, and intervention. While clinically important features may occur outside social interactions (e.g., genetics derived from intra-individual mechanisms), we restrict our discussion to social or interactional phenotypes within psychiatric and neurodevelopmental conditions. Using ASD as a primary example, I outline how MAGT can generate interpretable behavioral phenotypes, uncover mechanistic insights, and guide the design of adaptive, interaction-based interventions.

## 2 The limits of current frameworks

Despite substantial progress in cognitive neuroscience and psychiatry, prevailing frameworks for studying social dysfunction remain constrained by assumptions that limit their ability to capture real-world interaction. These limitations are particularly evident in conditions such as ASD and schizophrenia, where core symptoms unfold not in isolation but through ongoing exchanges with others.

### 2.1 Individual-centric models of social cognition

Classical theories of social cognition—including Theory of Mind (ToM), weak central coherence, executive dysfunction, and the social motivation hypothesis—have provided important conceptual scaffolds for understanding psychiatric disorders (25, 29–33). These frameworks have clarified how individuals may differ in mental-state attribution, information integration, cognitive control, and sensitivity to social reward. In ASD, ToM-based accounts emphasize difficulties in inferring beliefs and intentions (**Box 1**), whereas social motivation theories highlight the reduced salience of social stimuli. In schizophrenia, related constructs have been invoked to explain impairments in belief inference, aberrant salience, and disturbances in self-other distinction.

Box 1 **From theory of mind to multi-agent inference.**

Theory of Mind (ToM) has long served as a central framework for understanding social cognition, referring to the ability to attribute beliefs, intentions, and emotions to others (64). In autism research, ToM has been widely used to explain difficulties in interpreting social cues and predicting others’ behavior (65, 66). Classical experimental paradigms typically assess ToM using static or one-shot tasks, such as false-belief tests, that evaluate whether an individual can infer another’s mental state under simplified conditions.

However, real-world social interaction is not a one-time inference problem. It is a dynamic, reciprocal, and adaptive process in which agents simultaneously infer, act, and update their beliefs about one another. In this context, ToM represents only one component of a broader computational problem: how agents dynamically model and respond to other agents over time. Multi-agent inference extends ToM by embedding belief attribution within an interactive and iterative framework (67, 68). Rather than asking whether an individual can infer another’s belief, this perspective asks how agents: (i) update beliefs across repeated interactions, (ii) anticipate others’ strategies under uncertainty, (iii) adapt to changing behaviors and contexts, and (iv) coordinate toward (or fail to reach) stable patterns of interaction. In this view, social difficulties in ASD may reflect not only differences in mental state attribution, but also differences in belief updating, uncertainty representation, and strategic adaptation. For example, an individual may accurately infer another’s intention in a single instance, yet differ in how they generalize across interactions, weigh prior expectations, or respond to unpredictability.

Yet these models are fundamentally intra-individual. Social dysfunction is conceptualized as a deficit within a single agent, typically assessed through tasks that isolate specific cognitive components. While such reductionist approaches have been instrumental in identifying mechanisms, they treat interaction as a downstream consequence rather than a primary object of study. As a result, they offer limited insight into how behavior emerges in reciprocal, adaptive contexts, where outcomes depend on the coupling between multiple agents.

### 2.2 Experimental paradigms and the ecology gap

A central limitation of current approaches lies in the experimental paradigms used to operationalize social cognition. Much of the field relies on inherently non-interactive designs, including one-shot decision-making tasks, passive observation of social stimuli, or isolated responses to predefined scenarios. Although these paradigms afford experimental control, they systematically remove the defining features of social interaction: reciprocity, feedback, and mutual adaptation.

This over-reliance on static paradigms creates a fundamental mismatch between laboratory measurement and real-world social behavior. In natural settings, social interaction is continuous, adaptive, and shaped by bidirectional influence—each agent’s behavior dynamically alters the environment of the other, rendering the system inherently non-stationary. In contrast, many laboratory tasks capture only snapshots of behavior, precluding iterative belief updating and strategic adjustment.

This gap is particularly consequential for ASD and schizophrenia. In ASD, core difficulties often arise during sustained interactions—such as coordinating attention, maintaining turn-taking, and adapting to changing social cues—phenomena that cannot be captured in one-shot tasks. In schizophrenia, disturbances in belief formation and attribution may unfold over repeated exchanges, in which misinterpretations accumulate and stabilize into maladaptive interaction patterns. Static paradigms, by design, fail to capture these evolving dynamics.

### 2.3 Neglect of interactional dynamics

Current frameworks also underrepresent reciprocity, coordination, and mutual prediction in social behavior. Interaction entails alignment processes that unfold over time, including shared attention, negotiation of intent, and the stabilization of cooperative or competitive patterns. These processes are inherently relational and often nonlinear.

By focusing on individual-level variables, existing approaches struggle to formalize these dynamics. For example, social differences in ASD may reflect not only internal cognitive differences but also failures of coordination between agents with mismatched expectations or strategies. Similarly, in schizophrenia, altered belief updating may destabilize interaction patterns, contributing to mistrust, withdrawal, or pathological inference.

### 2.4 Limited integration across levels

A further limitation is the weak integration across behavioral, computational, and neural levels. Although neuroimaging and modeling have advanced considerably, they are often anchored to simplified tasks that fail to capture real interaction, constraining translation between neural mechanisms and naturalistic behavior.

At the same time, heterogeneity in psychiatric conditions remains difficult to interpret within existing frameworks. Variability is often treated as noise or reduced to symptom clusters, rather than understood as differences in underlying computational processes expressed during interaction. Without models that capture how individuals with distinct priors and learning dynamics engage with others, context-dependent variability remains poorly explained.

Taken together, these limitations point to a deeper conceptual gap: current frameworks lack a formal language for describing behavior as a property of coupled systems rather than isolated agents. They excel at decomposing cognition into components but fall short in explaining how these components are deployed and coordinated in dynamic social environments. Consequently, key phenomena—such as breakdowns in reciprocity (34), instability of shared understanding, and context-dependent variability—remain insufficiently formalized. Addressing this gap requires a shift in perspective: from individual cognition to interacting systems, in which behavior emerges through adaptation, mutual influence, and dynamic coordination among agents.

## 3 Multi-agent game theory as a unifying framework

### 3.1 A conceptual shift: social behavior as a multi-agent system

Addressing the limitations of current psychiatric frameworks requires a shift from individual-centric models to an interaction-based perspective (**Figure 1**). Social behavior does not arise within isolated individuals but through continuous exchange between agents. Rather than viewing social cognition as an internal computation, it is more naturally understood as *interactive inference*—a process in which individuals act, observe, and update beliefs in response to others. From this perspective, behavior emerges from coupled systems shaped by dynamic interdependence.

**Figure 1:**
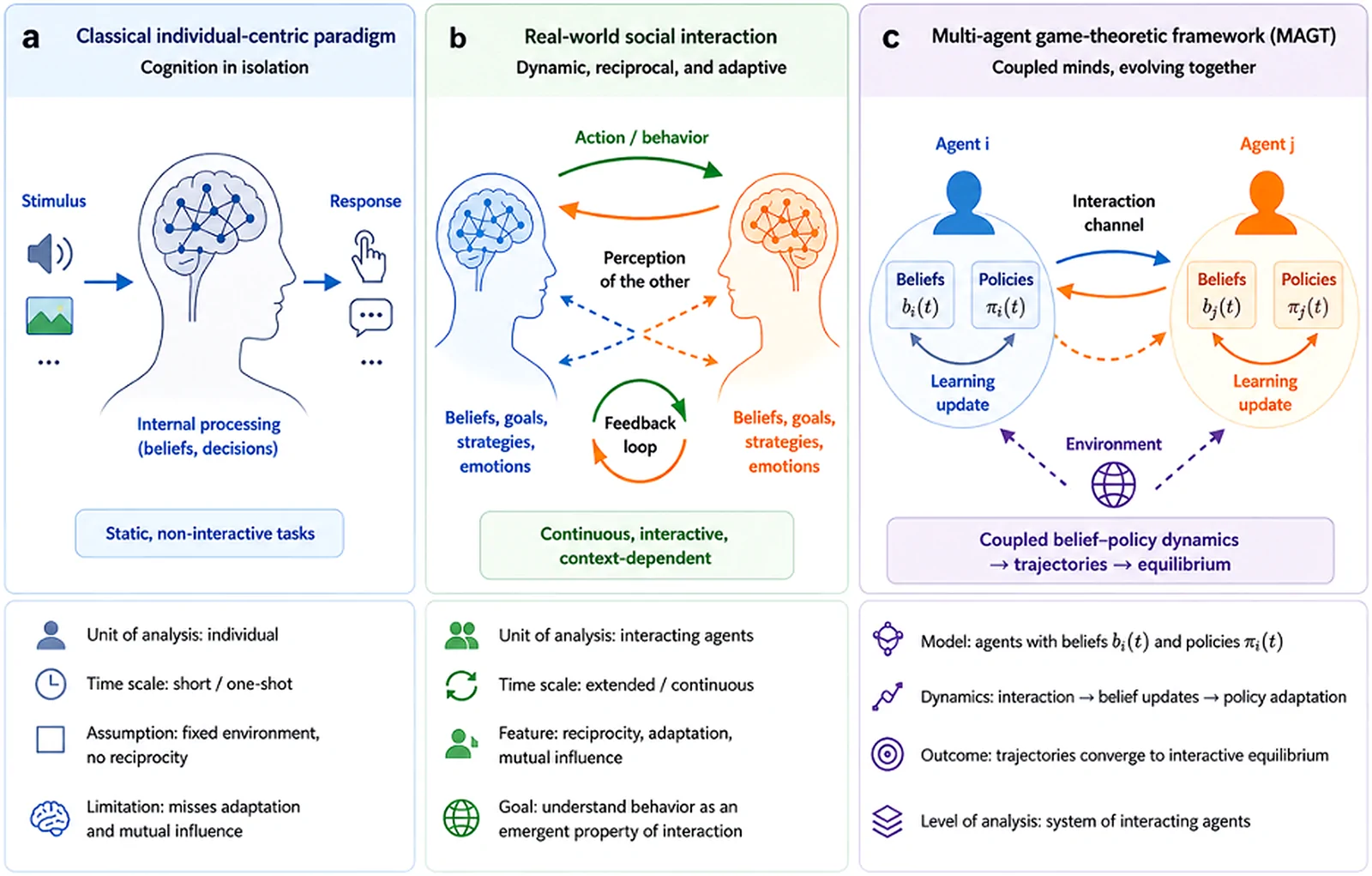
From individual cognition to multi-agent interaction: a conceptual shift. **(a)** Classical individual-centric paradigms conceptualize cognition within a single agent, where behavior is modeled as a stimulus–response process driven by internal computations (e.g., beliefs, decisions) under static, non-interactive task conditions. These approaches typically assume a fixed environment and neglect reciprocity and adaptation. **(b)** Real-world social interaction is inherently dynamic, reciprocal, and adaptive. Agents continuously perceive, act, and update their internal states in response to one another, forming closed feedback loops. Behavior emerges from ongoing exchanges shaped by beliefs, goals, and strategies, highlighting the importance of mutual influence and temporal context. **(c)** A multi-agent game-theoretic (MAGT) framework formalizes social interaction as a system of coupled agents. Each agent maintains time-evolving beliefs {*b_i_*(*t*), *b_j_*(*t*)} and policies {*π_i_*(*t*), *π_j_*(*t*)}, which are updated through interaction and learning within an environment. These coupled belief-policy dynamics generate trajectories that may converge to (or fail to reach) an interactive equilibrium. Social dysfunction can thus be understood as disruptions in coordination and alignment within this multi-agent dynamical system.

This reframing enables a critical shift in psychiatric inquiry: from asking *what is impaired within an individual* to *how interacting agents fail to coordinate*. In conditions such as ASD and schizophrenia, dysfunction may arise not only from internal deficits but from mismatches in beliefs, expectations, or learning dynamics between agents. These mismatches can destabilize interaction, preventing the formation of shared representations and coordinated behavior.

### 3.2 A formal framework for interaction

Multi-agent game theory (MAGT) provides a principled framework for formalizing these dynamics. Through the lens of games, it models real-world social behavior (including cooperation, competition, and negotiation) as structured interaction within environments defined by rules, actions, and consequences. Unlike individual-level approaches, outcomes depend on mutual adaptation: each agent’s behavior both shapes and is shaped by others. This interdependence gives rise to emergent patterns—cooperation, competition, coordination, or breakdown—that cannot be reduced to single-agent properties (35).

Within MAGT, interaction is described through a set of computational primitives: agents maintain beliefs about others, select policies, and evaluate outcomes through payoff structures encoding preferences such as reward or fairness (**Box 2**). Over repeated interaction, these processes may converge toward—or fail to achieve—stable configurations (“equilibria”), providing a mechanistic account of how social behavior unfolds over time.

Box 2 **Glossary of MAGT.**

Agent: A player or an autonomous entity that perceives its environment and acts.

Payoff: The reward an agent receives based on the combined actions of all players.

Policy: The strategy or rulebook an agent used to decide its next action based on the current state.

Belief: Perceived probability of specific latent action or policy of other players.

Equilibrium: A stable state where no agent has an incentive to change their strategy (behavioral equilibrium), policy or belief (cognitive equilibrium), or has reached neural synchrony among players (neural equilibrium).

Learning rule: The mathematical algorithm an agent uses to update its strategy based on past experiences and rewards. The tuning step-size parameter that determines how much new information overrides old information is called the learning rate.

Take the two-player Trust Game as an example. Imagine that Player A has $10 and can send some to Player B. The money sent triples on the way. Player B then decides how much to send back. The payoff is the final cash each player keeps after both players make their moves. The player’s game plan is referred to as policy. An equilibrium state is reached when neither player wants to change their strategy because they cannot get a better return.

Classical game theory has traditionally relied on simplifying assumptions, including perfect rationality, complete knowledge of the game structure, and symmetry across agents (7, 36), yet newer developments in game theory also accommodate settings of bounded rationality, asymmetry, and incomplete information. MAGT further relaxes these assumptions and accommodates partial observability, uncertainty, state switching, polyadic interaction, and heterogenous priors—particularly in psychiatric populations, in which cognitive processes, prior beliefs, and learning strategies can differ substantially across individuals. Additionally, agents differ in experience, cognitive constraints, and interpretations of social signals and must infer hidden states under noisy and evolving conditions. Incorporating these features allows MAGT to capture the variability, instability, and context dependence characteristic of disorders such as ASD and schizophrenia.

### 3.3 Multilevel interactive equilibrium

A key extension of this framework is multilevel interactive equilibrium (MIE), which links behavior, cognition, and neural dynamics (37). MIE is defined as a *joint fixed point* across levels or a multilevel consistency condition. Rather than defining equilibrium solely at the behavioral level (e.g., stable patterns of cooperation or competition), MIE posits alignment across multiple levels of the system: stable interaction patterns (behavioral), convergent beliefs or strategies (cognitive), and coordinated neural activity (neural). These levels are interdependent, such that disruption at one level can propagate across the system. A formal theoretical formulation of MIE is presented elsewhere (37). Consider a trust game: if two agents (an investor and a trustee) use stable and correlated behavioral policies (such as the proportion of money invested or returned), this may suggest a behavioral equilibrium. However, behavioral coordination alone does not necessarily imply convergence of beliefs or neural alignment. In an interactive partially observable Markov decision process (I-POMDP), agents may need to model the other player’s beliefs and use probabilistic beliefs to determine their actions; convergence of mutual belief estimates implies cognitive equilibrium. Furthermore, interpersonal neural synchrony or convergence to “neural attractors” implies neural equilibrium, which may arise sooner than the cognitive or behavioral equilibrium, as observed in computer simulations (Chen, unpublished data). The MIE framework also generates experimentally testable hypotheses and may predict how latent variables (such as the relative magnitude of acute stress) alter the time to equilibrium convergence or the temporal gaps among equilibria at different levels. To date, however, MIE remains a proposed theoretical framework that was studied primarily through computer simulations, and its clinical applications remain to be validated.

In psychiatric conditions, from the MIE viewpoint, social dysfunction may reflect a failure to achieve or sustain such equilibrium, manifesting as unstable coordination, misaligned expectations, or maladaptive interaction patterns. By integrating formal structure with realistic assumptions and multilevel dynamics, MAGT provides a unified framework for understanding how social behavior emerges, adapts, and breaks down in psychiatric disorders involving international deficits.

Furthermore, it is important to note that real-life social interactions do not necessarily converge to a stable state and may often involve temporary coordination and repeated switching between states or policies, resulting in persistent divergence from equilibrium. Nevertheless, MAGT remains useful for understanding non-equilibrium dynamics and analyzing or predicting complex, out-of-equilibrium behavior.

## 4 Social games as experimental platforms

A central challenge in psychiatry is to move from descriptive behavioral assessments to mechanistic understanding. Traditional paradigms often capture *what* individuals do, but offer limited insight into *how* and *why* those behaviors arise. Social games address this gap by embedding behavior within structured, interactive environments that are both experimentally controllable and computationally tractable (38).

Game-based paradigms—such as the ultimatum, trust, cooperation, and coordination tasks—have long been used to study decision-making under interdependence (20, 39). Their power is substantially enhanced when paired with adaptive agents, including algorithmic or AI-driven partners whose behavior can be systematically manipulated. These agents enable precise control over key dimensions of interaction, such as predictability, cooperation, strategic complexity, timing, and uncertainty, allowing researchers to probe responses to diverse social contingencies. Importantly, such designs can flexibly incorporate human–human, human–machine, or hybrid multi-agent interactions while maintaining ecological relevance.

A key strength of social games is their ability to generate dynamic, high-resolution measurements. Rather than yielding single outcomes, these paradigms capture trajectories of interaction over time—how individuals update beliefs, adapt strategies, and respond to feedback across repeated exchanges. The resulting behavioral time series provide a rich characterization of learning, adaptation, and coordination processes that cannot be accessed through static tasks. These trajectories can be further analyzed with computational models to extract interpretable parameters, including learning rates, sensitivity to reward or fairness, expectations about others, and exploration–exploitation trade-offs. Such parameters transform observable behavior into quantitative descriptors of underlying processes, linking action to mechanism.

Within this framework, we define “computational phenotypes of social interaction” as low-dimensional, interpretable representations of how individuals perceive, learn, and act in interactive environments (40). Unlike traditional phenotypes based on symptom scores or task accuracy, these phenotypes capture the *process* of interaction—how behavior unfolds over time and adapts to context—rather than merely its *outcome* (**Box 3**). They are inherently sensitive to partner characteristics, environmental structure, and uncertainty, making them particularly well suited for studying conditions such as ASD and schizophrenia, in which behavior is highly context dependent. Furthermore, in contrast to passive digital phenotyping (41, 42), social games actively probe latent interactional processes through controlled perturbations.

Box 3 **Computational phenotypes from social games.**

Social games provide a framework for deriving computational phenotypes for psychiatric disorders. In multi-agent settings—such as cooperation, negotiation, or competition tasks—behavior unfolds over time as individuals interact with other human or artificial agents, allowing researchers to move beyond outcome-based measures and instead characterize the processes that generate behavior. Within a game-theoretic framework, individual behavior can be decomposed into interpretable parameters, including:

• Belief dynamics: how individuals infer and update expectations about others’ actions.

• Learning rates: how quickly behavior adapts in response to feedback.

• Payoff sensitivity: how individuals value reward, fairness, or social reciprocity.

• Exploration-exploitation tradeoff: how individuals balance between trying new strategies and exploiting known ones.

• Uncertainty tolerance: how variability and unpredictability influence decision-making.

Collectively, these parameters may define a multidimensional space of interactional phenotypes, revealing structure that is not captured by diagnostic categories alone.

Crucially, computational phenotypes derived from social games are (i) mechanistically grounded, linking behavior to underlying cognitive processes; (ii) context-sensitive, capturing how behavior changes across environments and partners; (iii) quantitative and scalable, enabling comparison across individuals and populations; and (iv) translatable, as similar paradigms can be implemented in laboratory, clinical, and digital environments. In precision psychiatry, these computational phenotypes grounded in interaction dynamics can be combined with neuroimaging, physiological measures, and longitudinal data for helping clinical decision.

By combining controlled experimental design with computational modeling, social games enable a shift from measurement to mechanism. They provide a scalable platform for linking behavior to cognitive processes, and ultimately to neural dynamics, laying the foundation for a more precise and mechanistic science of social behavior in psychiatry.

## 5 NeuroAI and mechanistic bridges

A key challenge for precision psychiatry is to bridge abstract computational models of social interaction with the underlying neural mechanisms that give rise to behavior. While MAGT provides a formal description of interaction dynamics, its translational impact depends on linking these dynamics to brain systems. Advances in NeuroAI offer a promising pathway by integrating neuroscience, machine learning, and dynamical systems theory (43, 44).

Emerging evidence suggests that social interaction is supported not only by within-brain processes but also by coupled dynamics across brains. Studies of brain-to-brain coupling show that interacting individuals exhibit temporally aligned neural activity, particularly in regions associated with social cognition, attention, and action coordination (45, 46). These findings suggest that social behavior is inherently distributed across agents rather than confined to a single brain. In this view, disorders such as ASD and schizophrenia may involve an impaired ability to establish or sustain such inter-brain alignment, leading to instability in shared representations and coordination.

At the circuit level, social interaction engages distributed networks spanning prefrontal, temporal, and limbic systems (47). The prefrontal cortex (PFC) supports strategic reasoning and policy selection (48), temporal regions contribute to social perception and the representation of others’ states, and limbic structures encode salience and affective value. Critically, these systems operate in concert to enable inference, prediction, and adaptation in real time. A key objective is to map these circuit-level processes onto computational constructs central to MAGT, including belief updating (PFC), prediction error and conflict monitoring (anterior cingulate cortex) (49), mentalizing (temporoparietal junction) (50), payoff evaluation (orbitofrontal cortex and ventral striatum), and equilibrium formation.

Bridging these levels requires a new class of models. MARL provides a natural framework for capturing non-stationarity, feedback, and strategic co-adaptation in repeated interaction (51). In parallel, recurrent neural network (RNN)–based agents offer flexible architectures for representing internal states, memory, and temporal dependencies. Trained on social learning or interaction tasks (52, 53), such models can reveal emergent representations and strategies that parallel those observed in human behavior, providing a computational testbed for linking theory to mechanism.

A particularly promising direction lies in identifying shared latent subspaces across interacting agents. During interaction, internal representations—whether neural or artificial— may partially align within low-dimensional manifolds that support coordination and prediction. Recent work suggests that such shared representations can be identified in both neural recordings and trained artificial systems (54, 55), offering a quantitative measure of inter-agent alignment. In this context, social dysfunction may reflect failures to establish or stabilize these shared subspaces, resulting in misaligned beliefs, expectations, or actions. Although some promising evidence has been reported in animal studies, limited data are available from human studies. However, shared or partially aligned neural representations are not necessarily evidence of more successful social interaction; reduced inter-brain coupling or neural alignment in patients with ASD or schizophrenia patients remains a hypothesis requiring future clinical testing.

Finally, causal perturbation approaches such as noninvasive neurostimulation, combined with hyperscanning, provide a route to testing these mechanisms (56). Closed-loop systems incorporating neurofeedback or stimulation enable targeted manipulation of interaction dynamics, allowing researchers to probe how perturbations at one level propagate across agents and scales. Together, these advances support a unifying view of social behavior as a system of coupled dynamical processes operating across agents and levels. MAGT defines the structure of interaction, while NeuroAI provides the tools to map this structure onto neural dynamics and learning mechanisms. Integrating these perspectives moves the field beyond descriptive accounts toward mechanistic explanations that link brain, behavior, and interaction. Collectively, this framework opens new avenues for identifying neural signatures of interactional phenotypes and designing interventions that target not only individuals, but the dynamics of coordination between them.

## 6 Toward precision psychiatry

A central promise of precision psychiatry is to move beyond coarse diagnostic categories toward mechanistically grounded, individualized characterization and intervention. A multi-agent, game-theoretic perspective provides a natural path by redefining phenotypes in terms of how individuals behave, learn, and adapt within interactive environments. Rather than static traits or symptom checklists, this framework emphasizes interactional processes that are measurable, computationally interpretable, and directly linked to real-world social function.

### 6.1 Computational phenotyping from social interaction

Social games offer a powerful substrate for deriving candidate computational phenotypes. Unlike traditional measures that summarize outcomes, game-based paradigms capture trajectories of interaction—how individuals update beliefs, respond to feedback, and adjust strategies over time (57). These dynamics can be decomposed into interpretable parameters, including individualized learning rates, sensitivity to social reward or fairness, tolerance of uncertainty, and tendencies toward cooperation or avoidance. Although these parameters can vary across tasks, interaction partners, and contexts, systematically manipulating these behavioral conditions in controlled human-AI games may improve parameter identifiability and help distinguish parameters across conditions.

Such social game-derived features have several advantages (**Box 3**). These computational features are mechanistically interpretable and inherently context-sensitive, capturing how behavior adapts to different partners and environments; their generalizability across contexts can also be evaluated systematically. This is particularly important in conditions such as ASD and schizophrenia, in which behavior varies across contexts. Within a multi-agent framework, such measures define interactional phenotypes that provide a more precise characterization than traditional labels.

However, a substantial gap remains between computational modeling and psychiatric practice, in which behaviors are highly heterogeneous and context sensitive. Future study should characterize and quantify the within-subject, within-group, and between-group variability in these computational features. Although these parameters may represent useful candidate computational markers, their clinical interpretation still requires reproducible evidence of parameter recovery, reliability, validity, and stability across tasks and partners, as well as their ability to predict clinically meaningful outcomes.

### 6.2 From individual training to interaction design

A multi-agent perspective also reshapes interventions. Traditional approaches focus on training individuals in isolation, aiming to improve specific cognitive or behavioral skills. In contrast, if dysfunction arises from interactional dynamics, interventions can target the structure of interaction itself. This shift enables adaptive, technology-mediated environments in which social contingencies are systematically controlled and personalized. Virtual reality (VR) may provide a scalable and controllable platform for modeling and assessing social interactions (58, 59). For example, VR with adaptive agents or avatars can dynamically adjust predictability, difficulty, or feedback based on behavior. Similarly, human–AI interaction scaffolds can provide responsive partners that facilitate coordination, turn-taking, and joint attention. While still in its infancy, the development of such systems may support repeated, controlled interaction while preserving ecological validity, moving beyond one-size-fits-all toward closed-loop, personalized interventions.

### 6.3 Clinical integration across modalities

Realizing this vision requires integrating behavioral, neural, and physiological data to capture the full spectrum of interactional processes. Multi-agent paradigms can be combined with neuroimaging, electrophysiology, and wearable sensing to monitor brain activity, autonomic responses, and behavior during interaction. This enables computational parameters—such as belief updating or reward sensitivity—to be linked to underlying neural circuits and physiological states. For example, prefrontal, temporal, and limbic systems support strategic adaptation and social valuation, while physiological signals such as heart-rate variability and eye gaze index engagement and attention. Aligning these signals within a shared computational framework enables multilevel phenotyping that connects neural dynamics, behavioral strategies, and clinical outcomes.

### 6.4 Rethinking ASD through MAGT

Disruptions in social interaction are central to many psychiatric and neurodevelopmental conditions, most prominently ASD. In the view of “double empathy problem,” social dysfunction arises from mismatched bidirectional communication between different minds (or neurotypes) during interactions, rather than solely from a deficit within an autistic individual (60). Communication is reciprocal; an alternative yet complementary view is that deficits arise from difficulties in dyadic or polyadic interaction. Core difficulties associated with ASD in childhood and adolescence involve impaired reciprocity, joint attention, prediction of others, social motivation, and flexible adaptation (61, 62). These features emerge dynamically through live interactions between individuals, motivating a multi-agent account. Therefore, ASD may be framed as a failure of coordination or misalignment between players across multiple levels within the MAGT framework. Individuals may show slower belief updating, heightened sensitivity to unpredictability, or atypical valuation of social outcomes. From a dynamical systems viewpoint, uncoordinated equilibria may lead to impaired attentional engagement, and temporal misalignment may lead to disrupted turn-taking. From a learning or inference viewpoint, heterogeneity may reflect differences in priors, learning rates, and payoff structures that shape interaction across contexts.

An important issue concerning computational features derived from the MAGT framework is their interpretability in clinical practice. An essential question for precision psychiatry is how much of each estimated parameter (e.g., learning rate, reward sensitivity, or exploration-exploitation balance) reflects the individual, the partner, the dyad, or the task context. Although naturalistic human–human interaction experiments are valuable, standardized AI partners used in human–AI interactions provide a controllable and scalable platform for systematically manipulating partner behavior, context, and task difficulty and for further determining the variance in derived parameters explained by each factor. Although clinical examples are still lacking, it is reasonable to envision applying the same social games to examine human–human and human–AI interactions by varying their interaction partners, comparing differences in computational phenotypes and corelating these features with clinical, physiological, and neural measurements. Ultimately, these features could be used to evaluate treatment response, predict outcomes, or select intervention.

Finally, the theoretical equilibrium concept in the MIE proposal should be distinguished from a clinically desirable equilibrium. A social interaction can become stable while remaining maladaptive, rigid, or avoidant. Convergence to equilibrium or greater neural or behavioral alignment therefore should not automatically be interpreted as improvement. This distinction is important for ASD, in which the goal is not simply greater conformity to neurotypical interaction norms. In clinical practice, this problem may be framed as a patient–therapist interaction, in which the therapist’s policy can be customized to incorporate patient-valued interactional outcomes (e.g., “reaching equilibrium” vs. “escaping equilibrium,” depending on the context). Additionally, clinical interventions can be selected according to the desired in-equilibrium or out-of-equilibrium criterion.

## 7 Challenges and outstanding questions

Despite its promise, the MAGT framework faces challenges at theoretical, experimental, and translational levels. At the theoretical level, it remains unclear which equilibrium concepts are both realistic and scalable to complex, real-world social interactions. MIE offers a starting point but requires methods for estimation and validation, particularly in online and non-stationary settings in which sample sizes are limited. At the experimental level, progress depends on developing ecologically valid social-game paradigms that capture real-world interaction while remaining computationally tractable. Longitudinal and developmental designs will be essential for tracking how computational phenotypes evolve over time. At the translational level, linking deficits observed in social games to clinical outcomes demands rigorous validation alongside careful ethical consideration of measurement, interpretation, and intervention. Identifying clinically meaningful candidate computational markers requires systematic validation of parameter recovery, reliability, and stability across tasks and partners, as well as the prediction of clinical outcome.

Looking forward, many fundamental questions emerge. First, *how is social information represented*? Do individuals differ in how they encode others’ beliefs, intentions, and emotions or in how they assign salience to social versus non-social signals? Furthermore, how do mismatches between interacting agents shape behavior and interactional outcomes? How are individual parameters represented at the personalized level? Future studies that separate player, partner, and contextual effects by using multiple interaction partners and standardized artificial agents may help resolve these questions. Second, *how are prediction and uncertainty handled in social contexts?* Social interaction requires continuous anticipation and adaptation. Computationally, are individual differences best captured by altered learning rates, atypical representations of uncertainty, or reduced generalization across contexts? How does uncertainty vary across different interactional environments? It is important to identify measures that are both context-sensitive and stable to ensure clinical viability. Third, *how can neural mechanisms be linked to behavior?* Which circuits and large-scale network dynamics support social inference and coordination, and how are they altered in specific disorders (63)? How are others’ beliefs represented in the brain? How do neural processes support convergence (or failure to converge) toward stable interactional states? Future experiments combining game behavior with neuroimaging, EEG hyperscanning, and causal perturbation may provide some answers. Lastly, *how can theory inform intervention?* If social dysfunction reflects differences in learning, inference, or coordination, can we design adaptive environments or assistive technologies that reshape these processes? A key challenge is defining what constitutes a meaningful improvement in social interaction, particularly within neurodiversity-informed frameworks.

Social behavior has traditionally referred to naturalistic human-human interactions. However, human-AI interactions have emerged as a powerful tool in psychiatry. In the context of social games, human partners are likely to capture more of the complexity of real-world social interaction, whereas AI partners may offer greater experimental control and manipulability despite lacking embodied emotion. The combined use of human-human and human-AI interactions in experimental designs, as well as demonstration of the ecological validity of AI partners, remains an important research topic.

Finally, applying MAGT to precision psychiatry raises ethical issues related to privacy, algorithmic bias, autonomy, behavioral manipulation, anthropomorphism in human-AI interaction, accessibility, and equity. A broader community must work together to reach consensus regarding the norms of desired interaction and the structure of reward, especially when the framework is applied to neurodevelopmental populations.

## 8 Conclusion

This Perspective argues for a shift from static, individual-level deficits to dynamic, interaction-level mechanisms. Although intentionally bold and forward-looking, the connection between MAGT and precision psychiatry remains largely conceptual. In this view, a multi-agent, game-theoretic approach provides a principled pathway toward precision psychiatry grounded in interaction. By deriving interpretable and context-sensitive computational phenotypes, informing adaptive, interaction-based interventions, and integrating multimodal data, this approach reframes psychiatric disorders involving interactional behavioral deficits as emergent properties of coupled systems rather than isolated impairments. Realizing this vision will require coordinated advances across theory, experimentation, and clinical translation. Integrating MARL, Bayesian inference, neuroimaging, and closed-loop intervention design offers a promising route toward mechanistic understanding. In conclusion, psychiatric research grounded in social interaction has the potential to redefine both diagnosis and intervention, advancing a more precise, dynamic, and actionable science of mental health.

## Data Availability

The original contributions presented in the study are included in the article/supplementary material. Further inquiries can be directed to the corresponding author.
